# The potential role of perceived neighborhood social cohesion on COVID-19 vaccination uptake among individuals aged 50 and older: Results from the Korean Community Health Survey

**DOI:** 10.1371/journal.pone.0312309

**Published:** 2024-10-22

**Authors:** Younhee Kim, Min Kyung Lim

**Affiliations:** Department of Social and Preventive Medicine, College of Medicine, Inha University, Incheon, South Korea; Universitas Syiah Kuala, INDONESIA

## Abstract

The COVID-19 pandemic highlighted the critical importance of vaccination in controlling infectious diseases. While previous research has identified social cohesion as a potential facilitator of health behaviors, empirical studies exploring its direct impact on COVID-19 vaccination rates, especially across different age groups, remain limited. This cross-sectional study utilized data from the 2021 Korean Community Health Survey, a nationally representative survey conducted in South Korea. The analysis focused on adults aged 50 and older, categorizing them into two age groups (50–64 and ≥65). We investigated the association between perceived neighborhood social cohesion and COVID-19 vaccination status, controlling for socio-economic status, health behaviors, and concerns related to COVID-19. Statistical analysis was conducted using complex sample multiple logistic regression to adjust for potential confounders. The study included 135,352 participants, with an analysis showing that in the age group ≥65, higher levels of perceived neighborhood social cohesion were significantly associated with increased vaccination uptake (aOR for trust between neighbors: 1.200, 95% CI: 1.058–1.362; aOR for mutual assistance among neighbors: 1.491, 95% CI: 1.312–1.695). Interestingly, these associations were not significant in the 50–64 age group. Additionally, satisfaction with healthcare services was associated with higher vaccination uptake in both age groups (aOR: 1.106, 95% CI: 1.004–1.219 for 50–64; aOR: 1.306, 95% CI: 1.160–1.471 for ≥65). Our findings suggest that perceived neighborhood social cohesion plays a crucial role in influencing COVID-19 vaccination uptake among older adults, particularly those aged 65 and above. These results indicate that health policies aimed at enhancing social cohesion may effectively improve vaccination rates, especially among the elderly. Future research should explore the impact of social cohesion on other age groups and assess the causal relationships in longitudinal studies.

## 1. Introduction

The COVID-19 pandemic, caused by severe acute respiratory syndrome coronavirus 2 (SARS-CoV-2), emerged as a global crisis in early 2020 [1]. To control the spread of this virus, non-pharmaceutical interventions (NPIs) such as social distancing, mask-wearing, handwashing, and quarantine were promptly implemented. Despite the effectiveness of these measures in mitigating the immediate spread, they were not sufficient for long-term control of the virus. The need for effective and reliable vaccination strategies was underscored for proactive and sustainable management, as vaccines are crucial not only in are crucial not only in reducing the incidence of cases but also in significantly reducing the risk of hospitalization and death [2–4] The United Kingdom was the first to initiate a COVID-19 vaccination program following the emergency use authorization of the Pfizer-BioNTech vaccine, with other countries including South Korea subsequently launching their vaccination campaigns [2, 5].Nonetheless, COVID-19 vaccination was the most potent and effective means of controlling and preventing COVID-19 [6, 7], COVID-19 vaccine hesitancy had emerged as a significant issue due to concerns about the side effect and effectiveness given the rapid development process [8–12]. Even before the COVID-19 pandemic, the World Health Organization recognized vaccine hesitancy as a major threat to global health and emphasized the need for strategies to enhance public trust and acceptance [13]. Although the pandemic has now transitioned into an endemic phase, the potential for future outbreaks of novel infectious diseases persists. This underscores the continued importance of vaccination campaigns and preparedness for future pandemics. Emergency authorization for vaccines may still be necessary to control new infectious diseases. Therefore, understanding the factors influencing vaccine hesitancy and promoting vaccination is crucial not only for controlling current outbreaks but also for preparing for future pandemics [14–21].

Social cohesion, defined as the degree of connectedness and solidarity among groups within a society, encompasses the perceived sense of community, willingness of neighbors to intervene for the common good, sense of belonging among residents, and the level of trust shared among neighbors [22, 23]. Research indicates that individuals in cohesive and socially integrated communities are more likely to engage in healthy behaviors and experience better health outcomes [24, 25]. This can be attributed to the rapid dissemination of information about health, higher likelihood of adopting healthy behavioral norms, informal social control of health-related deviant behaviors, and the provision of mutual support when necessary [23, 26]. While social cohesion is generally considered a community level characteristic[22, 25, 27], there are limitations to treating it as such due to differences in residents’ perceptions of neighborhood boundaries and variations in their perceptions of the level of neighborhood social cohesion. To address this, some studies examining the relationship between social cohesion and health outcomes have utilized individual-level measurements for social cohesion. In this study, to emphasize the measurement of neighborhood social cohesion at the individual rather than the community level, we use the term "perceived neighborhood social cohesion," as referenced in previous research [24, 28]. Perceived social cohesion has been reported to be associated with influenza vaccination uptake [28], and COVID-19 vaccination intention and uptake [29, 30]. Additionally, perceived social exclusion increased the likelihood of vaccine refusal [31]. These findings highlight the importance of enhancing social cohesion as a strategy to improve vaccination rates and public health outcomes.

However, empirical studies investigating the relationship between perceived neighborhood social cohesion and actual COVID-19 vaccination, rather than vaccination intent, are limited. Additionally, previous research has not sufficiently explored the influence of social cohesion across different age groups. Understanding the impact of neighborhood social cohesion on vaccination behaviors across various age groups is crucial, as differences in social dynamics and health perceptions by age can affect the effectiveness of public health interventions. Identifying these differences can inform the development of targeted strategies to improve vaccination rates and overall public health outcomes.

This study aims to investigate the association between perceived neighborhood social cohesion and COVID-19 vaccination uptake across different age groups. Utilizing a large-scale, nationally representative sample, we controlled for other covariates influencing vaccination rates.

## 2. Materials and methods

### 2.1. Data source

This study used data from the 2021 Korean Community Health Survey (KCHS). The KCHS is a nationwide survey conducted annually by the Korea Disease Control and Preventive Agency (KDCA) and managed as a nationally approved statistical data source (approval number 117075). The 2021 KCHS was conducted using household visits between August 16 and October 31, 2021. The survey targeted adults aged 19 and over, and was conducted by city, province, and housing type using resident registration data. Detailed information on the sample design and survey contents of the KCHS has been previously reported [32].

The KCHS questionnaire contains 163 questions in 18 areas of the individual and household surveys. The individual survey includes health behaviors such as smoking and alcohol consumption, health checkups and vaccination status within the past year (influenza and COVID-19 vaccines), morbidity, healthcare utilization, accidents, and poisonings (injuries), activity limitations and quality of life, education level and economic activity, and COVID-19–related items.

Given the initially limited supply of vaccines in 2021, the South Korean government established a prioritized vaccine distribution strategy, focusing first on high-risk groups. Beginning with long-term care facility residents and healthcare workers on February 26, 2021 those aged ≥ 65 and 50 to 64 years were vaccinated sequentially up to August 2021 [5] (S1 Fig). Therefore, considering the time frame of the KCHS, individuals aged ≥ 50 were likely to be unvaccinated against COVID-19 due to vaccine hesitancy rather than issues with the vaccine delivery schedule. On the other hand, those aged < 50 were likely to remain unvaccinated at that time due to the government’s vaccine delivery policy.

Therefore, this analysis was restricted to individuals aged ≥ 50 to avoid potential confounding factors, such as vaccine accessibility. In addition, we excluded recipients of basic livelihood assistance from the study populations. Of the 229,242 individuals who participated in 2021 KCHS, the data of 135,352 were used in this analysis.

### 2.2. Variable

The dependent variable in this study was COVID-19 vaccination status. In the KCHS, participants were asked whether they had received a COVID-19 vaccination and responded either “Yes” or “No”. In this context, a “Yes” response was categorized as an event. Considering the survey period, all respondents included in the study had received their first vaccine dose.

The perceived neighborhood social cohesion as independent variable was measured using two items used previously [33] namely, “People in our neighborhood can be relied upon and trusted” (hereafter referred to as “trust between neighbors”) and “People in our neighborhood assist each other during events like ceremonies.”(hereafter referred to as “mutual assistance among neighbors”). In addition, an additional variable was introduced to assess satisfaction with healthcare services in the neighborhood. For each of these items, responses were either “Yes” or “No”.

Further, the study included variables related to socioeconomic status, health behaviors, self–reported health, and concerns about COVID–19, all of which have been identified as associated with vaccination in previous studies [15–18, 34]. Socioeconomic status was assessed based on age, sex (male or female), educational level (less than high school or high school or above), monthly household income (below or above 3 million Korean won), and residence (urban or rural), and household composition (living alone or with family members). Health behaviors and self-reported health were evaluated using smoking status (non–smoker or current smoker), drinking frequency (less than once a month or once a month or more), physical activity (moderate or higher intensity at least 5 days a week or less than 5 days a week), experience of depression (whether experienced for two weeks or more during the previous year), and self-reported health (fair or poor versus good). Concerns related to COVID-19, such as those associated with infection risk, social stigma, and economic damage due to the pandemic, were also considered. These concerns were categorized into three levels: high concern (very much so or somewhat), moderate concern, and low or no concern (not really or not at all).

### 2.3. Statistical analysis

The analysis was conducted considering weights, stratification variables, and clustering variables to reflect the complex sample design accurately. In the descriptive analysis, baseline characteristics were presented as frequencies and weighted percentages for categorical variables and as weighted means and standard errors for continuous variables. Adjusted odds ratios (aORs) and 95% confidence intervals (CI) of factors associated with COVID-19 vaccination, after adjusting for all covariates used in the study, were analyzed using complex sample multiple logistic regression analysis. Analyses were performed separately on participants aged 50–64 and 65 and over.

Data processing and statistical analyses were performed using SAS version 9.4 (SAS Institute, Cary, NC, USA), and ‘proc survey’ procedures were used for complex stratified cluster analysis.

## 3. Results

The baseline characteristics of the 135,352 participants by age group are presented in Table 1. The 50–64 age group included 66,244 individuals, and the ≥ 65 group 69,108 individuals. In the 50–64 age group, 69.79% expressed trust between neighbors, 52.51% mutual assistance among neighbors, and 75.27% satisfaction with healthcare services in their community. However, in the ≥ 65 age group, these proportions were 79.52%, 69.44%, and 78.85%, respectively, indicating higher levels of perceived neighborhood social cohesion and satisfaction with community healthcare services. In addition, members of the ≥ 65 age group engaged in smoking, drinking, and physical activity less frequently than those in the 50–64 age group.

**Table 1:** Baseline characteristics of the participants.

	Total	50–64 years old	65 years old or older
	n (weighted %)	n (weighted %)	n (weighted %)
	or mean ± SE	or mean ± SE	or mean ± SE
Total	135,352 (100.00%)	66,244 (48.94%)	69,108 (51.06%)
Sex			
Male	59,901 (44.26%)	30,342 (45.80%)	29,559 (42.77%)
Female	75,451 (55.74%)	35,902 (54.20%)	39,549 (57.23%)
Age (yrs.)	63.65±0.04	56.66±0.02	73.76±0.03
Residence			
Urban	64,526 (47.67%)	36,498 (55.10%)	28,028 (40.56%)
Rural	70,826 (52.33%)	29,746 (44.90%)	41,080 (59.44%)
Education level			
<High school	67,958 (50.21%)	17,008 (25.67%)	50,950 (73.73%)
≥High school	67,282 (49.71%)	49,172 (74.23%)	18,110 (26.21%)
Income (monthly)			
<3 Korean million won	78,651 (58.11%)	24,674 (37.25%)	53,977 (78.11%)
≥3 Korean million won	56,701 (41.89%)	41,570 (62.75%)	15,131 (21.89%)
Income (Korean million won)	371.95 ± 1.6	465.88 ± 2.18	236.26 ± 1.42
Household composition			
Living with family members	110,591 (81.71%)	58,196 (87.85%)	52,395 (75.82%)
Living alone	24,761 (18.29%)	8,048 (12.15%)	16,713 (24.18%)
Smoking status			
Non–smoker	117,433 (86.76%)	54,059 (81.61%)	63,374 (91.70%)
Smoker	17,917 (13.24%)	12,184 (18.39%)	5,733 (8.30%)
Alcohol consumption (times/month)			
<1	88,718 (65.55%)	35,377 (53.40%)	53,341 (77.18%)
≥ 1	46,623 (34.45%)	30,864 (46.59%)	15,759 (22.80%)
Physical activities			
No	46,625 (34.45%)	30,865 (46.59%)	15,760 (22.80%)
Yes	46,627 (34.45%)	30,866 (46.59%)	15,761 (22.81%)
Experience of depression			
No	46,629 (34.45%)	61,906 (93.45%)	64,101 (92.75%)
Yes	46,631 (34.45%)	4,336 (6.55%)	4,990 (7.22%)
Self–reported health			
fair or poor	88,934 (65.71%)	38,509 (58.13%)	50,425 (72.97%)
good	46,415 (34.29%)	27,734 (41.87%)	18,681 (27.03%)
Trust between neighbors			
Trust	101,189 (74.76%)	46,233 (69.79%)	54,956 (79.52%)
No trust	34,123 (25.21%)	19,980 (30.16%)	14,143 (20.47%)
Mutual assistance among neighbors			
Assistance	82,774 (61.15%)	34,782 (52.51%)	47,992 (69.44%)
No assistance	52,558 (38.83%)	31,448 (47.47%)	21,110 (30.55%)
Satisfaction with healthcare service in community			
Satisfaction	104,356 (77.10%)	49,861 (75.27%)	54,495 (78.85%)
No satisfaction	30,988 (22.89%)	16,377 (24.72%)	14,611 (21.14%)
Concern about infection			
High	88,245 (65.20%)	43,018 (64.94%)	45,227 (65.44%)
Moderate	27,079 (20.01%)	14,817 (22.37%)	12,262 (17.74%)
Low or no	20,016 (14.79%)	8,406 (12.69%)	11,610 (16.80%)
Concern about social stigma			
High	102,472 (75.71%)	49,419 (74.60%)	53,053 (76.77%)
Moderate	17,234 (12.73%)	9,096 (13.73%)	8,138 (11.78%)
Low or no	15,610 (11.53%)	7,717 (11.65%)	7,893 (11.42%)
Concern about economic damage			
High	106,241 (78.49%)	50,847 (76.76%)	55,394 (80.16%)
Moderate	14,921 (11.02%)	8,013 (12.10%)	6,908 (10.00%)
Low or no	14,178 (10.47%)	7,384 (11.15%)	6,794 (9.83%)

Values are presented as unweighted numbers (weighted %)

The characteristics of individuals by vaccination status and the results of the multiple regression analysis are presented in Table 2. In addition, crude odds ratios from the univariate analysis are shown in S1 Table. The crude odds ratios calculated from the univariate analysis of the relationship between vaccination status and each covariate did not differ significantly from the adjusted odds ratios in the multiple regression analysis. Vaccination rates were lower among males than females in the 50–64 age group (92.24% vs. 93.37%), while they were higher among males in the ≥ 65 group (95.21% vs. 94.82%). Participants residing in urban areas, with a higher level of education, higher household income, drinking more frequently, and more engaged in physical activity were more likely to be vaccinated, whereas those living alone, smokers, those who experienced depression, and those who self-rated their health as good were less likely to be vaccinated. Concerns about infection, social stigma, and economic damage due to COVID-19 were positively correlated with vaccination rates, and participants who reported high levels of perceived neighborhood cohesion and satisfaction with healthcare services in their community were also more likely to be vaccinated.

**Table 2:** Vacciation status and the results of the complex sample multiple logistic regression analysis.

	50–64 years old		65 years old or older	
	Vaccination	aOR (95% CI) ^1)^	Vaccination	aOR (95% CI) ^1)^
	n (weighted %) or mean ± SE		n (weighted %) or mean (SE)	
Total	61,394 (92.81%)	-	65,798 (95.00%)	-
Sex				
Male	27,862 (92.24%)	1.000 (Reference)	28,154 (95.21%)	1.000 (Reference)
Female	33,532 (93.37%)	1.200 (1.095, 1.314) *	37,644 (94.82%)	1.038 (0.929, 1.160)
Age (yrs.)	56.76 ± 0.02	1.097 (1.085, 1.109) *	73.66 ± 0.04	0.965 (0.957, 0.973) *
Residence				
Urban area	33,993 (92.10%)	1.000 (Reference)	26,656 (94.32%)	1.000 (Reference)
Rural area	27,401 (93.75%)	1.191 (1.079, 1.313) *	39,142 (96.57%)	1.166 (1.039, 1.309) *
Education level				
<High school	15,811 (92.61%)	1.000 (Reference)	48,511 (94.93%)	1.000 (Reference)
≥High school	45,527 (92.86%)	1.166 (1.048, 1.297) *	17,240 (95.09%)	0.865 (0.764, 0.978) *
Income (monthly)				
<3 million won	22,358 (90.26%)	1.000 (Reference)	51,310 (94.75%)	1.000 (Reference)
≥3 million won	39,036 (93.87%)	1.652 (1.509, 1.809) *	14,488 (95.55%)	1.124 (0.985, 1.284)
Household composition				
Living with family members	54,180 (93.21%)	1.000 (Reference)	49,926 (95.07%)	1.000 (Reference)
Living alone	7,214 (89.27%)	0.800 (0.719, 0.891) *	15,872 (94.68%)	1.143 (1.006, 1.297) *
Smoking status				
Non–smoker	50,536 (93.56%)	1.000 (Reference)	60,421 (95.12%)	1.000 (Reference)
Smoker	10,858 (89.60%)	0.644 (0.580, 0.714) *	5,376 (93.69%)	0.594 (0.497, 0.710) *
Alcohol consumption (times/month)				
<1	32,572 (92.02%)	1.000 (Reference)	50,475 (94.31%)	1.000 (Reference)
≥ 1	28,819 (93.65%)	1.552 (1.424, 1.691) *	15,317 (97.09%)	1.837 (1.574, 2.144) *
Physical activities				
No	49,035 (92.66%)	1.000 (Reference)	56,116 (94.68%)	1.000 (Reference)
Yes	12,295 (93.50%)	1.122 (1.012, 1.243) *	9,571 (97.12%)	1.516 (1.258, 1.827) *
Depressive expression				
No	57,486 (93.03%)	1.000 (Reference)	57,486 (95.30%)	1.000 (Reference)
Yes	3,907 (89.85%)	0.748 (0.651, 0.860) *	3,907 (91.35%)	0.596 (0.515, 0.689) *
Self–reported health				
fair or poor	35,430 (92.10%)	1.000 (Reference)	47,731 (94.32%)	1.000 (Reference)
good	25,963 (93.75%)	1.202 (1.109, 1.303) *	18,065 (96.57%)	1.458 (1.291, 1.646) *
Trust between neighbors				
Trust	43,002 (93.18%)	1.000 (Reference)	52,562 (95.57%)	1.000 (Reference)
No trust	18,365 (92.13%)	1.017 (0.928, 1.115)	13,228 (93.48%)	1.200 (1.058, 1.362) *
Mutual assistance among neighbors				
Assistance	32,368 (93.17%)	1.000 (Reference)	46,041 (96.07%)	1.000 (Reference)
No assistance	29,013 (92.60%)	1.045 (0.951, 1.147)	19,751 (93.88%)	1.491 (1.312, 1.695) *
Satisfaction with healthcare service in community				
Satisfaction	46,343 (93.09%)	1.000 (Reference)	52,056 (95.30%)	1.000 (Reference)
No satisfaction	15,046 (91.65%)	1.106 (1.004, 1.219) *	13,740 (93.48%)	1.306 (1.160, 1.471)
Concern about infection				
Concern	40,035 (93.20%)	1.046 (0.922, 1.186)	43,181 (95.14%)	0.893 (0.761, 1.049)
Moderate	13,677 (92.40%)	1.000 (0.871, 1.148)	11,741 (95.53%)	1.140 (0.938, 1.387)
No concern	7,679 (91.66%)	1.000 (Reference)	10,869 (93.81%)	1.000 (Reference)
Concern about social stigma				
Concern	46,123 (93.36%)	1.344 (1.186, 1.522) *	50,785 (95.49%)	1.598 (1.345, 1.900) *
Moderate	8,309 (92.04%)	1.156 (0.997, 1.340)	7,711 (94.71%)	1.277 (1.020, 1.599) *
No concern	6,951 (90.64%)	1.000 (Reference)	7,281 (92.40%)	1.000 (Reference)
Concern about economic damage				
Concern	47,217 (92.88%)	0.968 (0.851, 1.102)	52,955 (95.37%)	1.276 (1.079, 1.509) *
Moderate	7,400 (92.81%)	1.021 (0.866, 1.205)	6,528 (94.57%)	1.096 (0.878, 1.368)
No concern	6,777 (92.40%)	1.000 (Reference)	6,307 (92.91%)	1.000 (Reference)

Note: adjusted odds ratio (aORs) and 95% confidence intervals (CIs) were derived by complex sample multiple logistic regression analysis

* P<0.05

Complex sample multiple logistic regression analysis results are provided in Table 2. In the 50–64 age group, factors such as being female, older (within the age group), residing in a rural area, a higher education level, higher household income, and living with family members were significantly associated with higher vaccination rates. Conversely, in the ≥ 65 group, being younger (within the group), living alone, and having a lower education level were significantly associated with higher vaccination rates.

In both groups, smoking (aOR 0.644, 95% CI 0.580–0.714; aOR 0.594, 95% CI 0.497–0.710, respectively) and experience of depression (aOR 0.748, 95% CI 1.0.651–0.860; aOR 0.596, 95% CI 0.515–0.689, respectively) were negatively associated with vaccination rate, while alcohol consumption (aOR 1.552, 95% CI 1.424–1.691; aOR 1.837, 95% CI 1.574–2.144, respectively), physical activity (aOR 1.122, 95% CI 1.012–1.243; aOR 1.516, 95% CI 1.258–1.827, respectively), and good self-reported health (aOR 1.202, 95% CI 1.109–1.303; aOR 1.458, 95% CI 1.291–1.646, respectively) were positively associated with vaccination rate.

In the 50–64 age group, perceived neighborhood cohesion, as assessed by trust between neighbors and mutual assistance among neighbors, was not significantly associated with vaccination uptake. However, in the ≥ 65 group, higher levels of perceived neighborhood cohesion were positively associated with vaccination uptake (aOR 1.200, 95% CI 1.058–1.362 for trust between neighbors; aOR 1.491, 95% CI 1.312–1.695 for mutual assistance among neighbors). In both age groups, satisfaction with community healthcare services was associated with higher vaccination rates (aOR 1.106, 95% CI 1.004–1.219; aOR 1.306, 95% CI 1.160–1.471, respectively). Furthermore, those concerned about social stigma were more likely to be vaccinated in the 50–64 and ≥ 65 groups (aOR 1.344, 95% CI 1.186–1.522; aOR 1.598, 95% CI 1.345–1.900, respectively), whereas concern about economic damage due to COVID-19 was only associated with vaccination in the ≥ 65 group (aOR 1.279, 95% CI 1.079–1.509).

## 4. Discussion

This study aimed to examine the relationship between perceived neighborhood social cohesion and COVID-19 vaccination status utilizing data from the KCHS (2021), a nationally representative survey conducted annually in South Korea. Notably, findings indicated that among individuals aged ≥ 65, higher perceived neighborhood social cohesion was positively associated with COVID-19 vaccination uptake. However, this association was not observed in the 50–64 age group. The study demonstrates that the relationship between perceived neighborhood social cohesion and vaccination depend on age.

Previous studies have presented several hypothetical mechanisms whereby neighborhood social cohesion might facilitate the use of preventive healthcare services [28]. First, cohesive neighborhoods are more efficient at disseminating information about vaccination procedures including vaccines appointment scheduling and less crowded vaccination sites. Second, cohesive neighborhoods can provide psychological support to individuals with anxiety and concerns about vaccines. Third, cohesive neighborhoods can help establish and maintain healthy norms among community members, and thereby reducing vaccine hesitancy. The results of this study suggest that these mechanisms may be particularly influential in the elderly. Although few studies have studied the relationship between neighborhood social cohesion and vaccination by age, one study suggested that the association between social relationships and vaccination intake increases with age. A study conducted in Germany on adults over 40 found that individuals aged 60–75 who received informational support were more likely to receive influenza vaccinations, while this association was not observed in those aged under 60 [35]. In addition, previous studies that examined relationships between social participation and self-reported health [36], social activities and health-related quality of life [37], and social engagement and experience of depression [38] also indicated that these association were stronger in individuals aged ≥ 65. In addition, a study conducted in the United States exploring quality of social interaction across age groups found that middle-aged adults had the lowest scores for social interaction, while older adults had high positive ratings of their social interaction [39]. These previous studies suggest that perceptions and attitudes towards social relationships and social cohesion are age-dependent and that the influence of these social relationships and social cohesion on health behaviors, such as COVID-19 vaccination, may also depend on age.

The analysis showed that satisfaction with healthcare services in community was associated with higher COVID-19 vaccination rates. This finding is consistent with previous research [17]. Interestingly, in the 50–64 and ≥ 65 age groups, relationships between age, education level, household composition, and vaccination rates showed opposite trends. In the 50–64 group, a higher age (within the group), higher educational attainment, and living with family members were associated with vaccination uptake, whereas in the ≥ 65 group, a lower age (within the group), lower educational attainment, and living alone were associated with vaccination uptake. In a study of the relationship between age and the use of preventive health services, it was observed that influenza vaccination rates increased with age, whereas the probability of cancer screening peaked at 63 [35]. The results of our study demonstrate a pattern similar to previous studies regarding the correlation between cancer screening rates and age. Higher vaccination rates among older age groups may be explained by the risk of fatal consequences associated with COVID-19 infection. However, beyond a certain age, underlying health conditions may prevent individuals from getting vaccinated. In addition, perceptions of the safety and efficacy of COVID-19 vaccinations may depend on age. Such findings suggest that various demographic factors can influence vaccination behaviors in complex ways.

This study also revealed that smoking was negatively associated with vaccination, whereas alcohol drinking was positively associated. A previous study reported negative relationships between influenza vaccination and these health behaviors (both smoking and alcohol drinking) [20]. Both behaviors are considered unhealthy, and thus, individuals who engage in these activities are generally less likely to use preventive health care services, such as health screenings and vaccinations. However, this study shows that higher levels of alcohol consumption were associated with increased COVID-19 vaccination uptake. The Korean government offered vaccination incentives by easing gathering restrictions, and since drinking often occurs in social settings in South Korea [40, 41], this finding suggests that individuals who drank more frequently were more likely to get vaccinated. This indicates that the observed positive correlation between alcohol consumption and vaccination uptake might be due to the unique societal impact of COVID-19 and the incentives offered to encourage vaccination.

The limitations of this study are as follows. First, individuals who did not receive a COVID-19 vaccination due to temporary health-related reasons or problems with vaccination scheduling were not considered, although adjustment was made for self-reported health conditions. In addition, although stable vaccine supply and vaccination implementation were reported for those over 50 at the time of the survey, people who could not get vaccinated at that time due to an accessibility problem but were vaccinated later were not considered. As of October 28, 2022, the first-dose vaccination rate for COVID-19 in Korea was reported to be 90.6% for those aged ≥ 80, 96.2% for those in their 70s, 97.6% for those in their 60s, and 98.0% for those in their 50s [42]. It was difficult to confirm that the partial increase in COVID-19 vaccination rate after the survey was due to aggressive governmental policies encouraging vaccination and the continuous provision of information. In addition, since COVID-19 vaccination supply policy varied by age, caution is needed when interpreting the relationship between age and vaccination uptake. Second, this study employed cross-sectional data, and thus, is limited in terms of its ability to establish causal relationships between vaccination and other factors. However, considering that trust between neighbors, mutual assistance among neighbors, and satisfaction with medical service in neighborhoods are generally stable factors, it is reasonable to infer that these factors had a significant impact on vaccination uptake.

## 5. Conclusion

This study provides evidence that perceived neighborhood social cohesion significantly influences COVID-19 vaccination uptake among older adults in South Korea, particularly those aged 65 years and older. Our findings underscore that higher levels of social cohesion are positively associated with increased vaccination rates in this demographic, although no such relationship was observed in the younger cohort aged 50–64 years. These results highlight the crucial role of community bonds and mutual trust in influencing the health behaviors of older adults.

The implications of these findings are significant for public health policy. They suggest that enhancing social cohesion could be a strategic approach to increasing vaccination rates, particularly among the elderly, who benefit most from such community support structures. Health policies that incorporate social cohesion initiatives, such as community engagement programs and support networks, could effectively reduce vaccine hesitancy and enhance overall public health security against infectious diseases.

This study proposes further research in several areas. First, the impact of perceived social cohesion on vaccine uptake in younger populations and children needs to be explored. Second, the mechanisms through which social cohesion affects health behaviors across different cultures and community settings require further investigation. Lastly, longitudinal studies are necessary to establish causal relationships and to assess the long-term effects of social cohesion on vaccination behaviors.

In conclusion, our research provides crucial insights into the interplay between perceived neighborhood social cohesion and public health interventions. It advocates for a holistic approach to health policy that considers the social structure of communities as a fundamental component in improving disease prevention efforts, particularly in vaccination campaigns.

## Supporting information

S1 Fig COVID-19 vaccination delivery schedule in South Korea. (PDF)

S1 Table COVID-19 Crude odds ratios from the univariate analysis. (DOCX)

## References

[1] Zhou P, Yang X-L, Wang X-G, Hu B, Zhang L, Zhang W, et al. A pneumonia outbreak associated with a new coronavirus of probable bat origin. Nature. 2020;579(7798):270–3. doi: 10.1038/s41586-020-2012-7 32015507 PMC7095418

[2] Singh P, Anand A, Rana S, Kumar A, Goel P, Kumar S, et al. Impact of COVID-19 vaccination: a global perspective. Front Public Health. 2023;11:1272961. doi: 10.3389/fpubh.2023.1272961 38274537 PMC10808156

[3] Viveiros-Rosa SG, Mendes CD, Farfán-Cano GG, El-Shazly M. The race for clinical trials on Omicron-based COVID-19 vaccine candidates: Updates from global databases. Narra J. 2022;2(3):e88. doi: 10.52225/narra.v2i3.88 38449904 PMC10914133

[4] Adil Mahmoud Yousif N, Tsoungui Obama HCJ, Ngucho Mbeutchou YJ, Kwamou Ngaha SF, Kayanula L, Kamanga G, et al. The impact of COVID-19 vaccination campaigns accounting for antibody-dependent enhancement. PLoS One. 2021;16(4):e0245417. doi: 10.1371/journal.pone.0245417 33886573 PMC8061987

[5] Nham E, Song JY, Noh JY, Cheong HJ, Kim WJ. COVID-19 Vaccination in Korea: Past, Present, and the Way Forward. Journal of Korean Medical Science. 2022;37(47). 10.3346/jkms.2022.37.e351 PMC9723191 36472087

[6] Wouters OJ, Shadlen KC, Salcher-Konrad M, Pollard AJ, Larson HJ, Teerawattananon Y, et al. Challenges in ensuring global access to COVID-19 vaccines: production, affordability, allocation, and deployment. The Lancet. 2021;397(10278):1023–34. doi: 10.1016/S0140-6736(21)00306-8 33587887 PMC7906643

[7] Hasan T, Beardsley J, Marais BJ, Nguyen TA, Fox GJ. The Implementation of Mass-Vaccination against SARS-CoV-2: A Systematic Review of Existing Strategies and Guidelines. Vaccines. 2021;9(4):326. doi: 10.3390/vaccines9040326 33915829 PMC8066252

[8] Park HK, Ham JH, Jang DH, Lee JY, Jang WM. Political Ideologies, Government Trust, and COVID-19 Vaccine Hesitancy in South Korea: A Cross-Sectional Survey. International Journal of Environmental Research and Public Health. 2021;18(20):10655. doi: 10.3390/ijerph182010655 34682401 PMC8536119

[9] Lazarus JV, Wyka K, Rauh L, Rabin K, Ratzan S, Gostin LO, et al. Hesitant or Not? The Association of Age, Gender, and Education with Potential Acceptance of a COVID-19 Vaccine: A Country-level Analysis. Journal of Health Communication. 2020;25(10):799–807. doi: 10.1080/10810730.2020.1868630 33719881

[10] Willis DE, Andersen JA, Bryant-Moore K, Selig JP, Long CR, Felix HC, et al. COVID-19 vaccine hesitancy: Race/ethnicity, trust, and fear. Clinical and Translational Science. 2021;14(6):2200–7. doi: 10.1111/cts.13077 34213073 PMC8444681

[11] Skafle I, Nordahl-Hansen A, Quintana DS, Wynn R, Gabarron E. Misinformation About COVID-19 Vaccines on Social Media: Rapid Review. J Med Internet Res. 2022;24(8):e37367. doi: 10.2196/37367 35816685 PMC9359307

[12] Noh Y, Kim JH, Yoon D, Choe YJ, Choe S-A, Jung J, et al. Predictors of COVID-19 booster vaccine hesitancy among fully vaccinated adults in South Korea: a nationwide cross-sectional survey. Epidemiology and Health. 2022:e2022061. 35914771 10.4178/epih.e2022061 PMC9754905

[13] World Health Organization (WHO). Ten threats to global health in 2019: World Health Organization; 2019. Available from: https://www.who.int/news-room/spotlight/ten-threats-to-global-health-in-2019

[14] MacDonald NE. Vaccine hesitancy: Definition, scope and determinants. Vaccine. 2015;33(34):4161–4. doi: 10.1016/j.vaccine.2015.04.036 25896383

[15] Alshurman BA, Khan AF, Mac C, Majeed M, Butt ZA. What Demographic, Social, and Contextual Factors Influence the Intention to Use COVID-19 Vaccines: A Scoping Review. International Journal of Environmental Research and Public Health. 2021;18(17):9342. doi: 10.3390/ijerph18179342 34501932 PMC8431323

[16] Bish A, Yardley L, Nicoll A, Michie S. Factors associated with uptake of vaccination against pandemic influenza: A systematic review. Vaccine. 2011;29(38):6472–84. doi: 10.1016/j.vaccine.2011.06.107 21756960

[17] Limbu YB, Gautam RK. The determinants of COVID-19 vaccination intention: a meta-review. Front Public Health. 2023;11:1162861. doi: 10.3389/fpubh.2023.1162861 37377544 PMC10291626

[18] Roy DN, Biswas M, Islam E, Azam MS. Potential factors influencing COVID-19 vaccine acceptance and hesitancy: A systematic review. PLOS ONE. 2022;17(3):e0265496. doi: 10.1371/journal.pone.0265496 35320309 PMC8942251

[19] Taylor S, Landry CA, Paluszek MM, Groenewoud R, Rachor GS, Asmundson GJG. A Proactive Approach for Managing COVID-19: The Importance of Understanding the Motivational Roots of Vaccination Hesitancy for SARS-CoV2. Front Psychol. 2020;11:575950. doi: 10.3389/fpsyg.2020.575950 33192883 PMC7604422

[20] Eiden AL, Barratt J, Nyaku MK. Drivers of and barriers to routine adult vaccination: A systematic literature review. Human Vaccines & Immunotherapeutics. 2022;18(6). doi: 10.1080/21645515.2022.2127290 36197070 PMC9746483

[21] Karafillakis E, Simas C, Jarrett C, Verger P, Peretti-Watel P, Dib F, et al. HPV vaccination in a context of public mistrust and uncertainty: a systematic literature review of determinants of HPV vaccine hesitancy in Europe. Human Vaccines & Immunotherapeutics. 2019;15(7–8):1615–27. 30633623 10.1080/21645515.2018.1564436 PMC6783136

[22] Kawachi I, Berkman LF. Social capital, social cohesion, and health. In: B. Berkman LF, Kawachi I, Glymour MM, editors: Oxford University Press; 2000 01 Mar 2015.

[23] Kim ES, Chen Y, Kawachi I, VanderWeele TJ. Perceived neighborhood social cohesion and subsequent health and well-being in older adults: An outcome-wide longitudinal approach. Health Place. 2020;66:102420. doi: 10.1016/j.healthplace.2020.102420 32905980 PMC7686282

[24] Ruiz M, Scholes S, Bobak M. Perceived neighbourhood social cohesion and depressive symptom trajectories in older adults: a 12-year prospective cohort study. Soc Psychiatry Psychiatr Epidemiol. 2018;53(10):1081–90. doi: 10.1007/s00127-018-1548-4 29915902 PMC6182502

[25] Ehsan A, Klaas HS, Bastianen A, Spini D. Social capital and health: A systematic review of systematic reviews. SSM—Population Health. 2019;8:100425. doi: 10.1016/j.ssmph.2019.100425 31431915 PMC6580321

[26] Moore S, Kawachi I. Twenty years of social capital and health research: a glossary. J Epidemiol Community Health. 2017;71(5):513–7. doi: 10.1136/jech-2016-208313 28087811

[27] McNeill LH, Kreuter MW, Subramanian SV. Social Environment and Physical activity: A review of concepts and evidence. Social Science & Medicine. 2006;63(4):1011–22. 16650513 10.1016/j.socscimed.2006.03.012

[28] Kim ES, Kawachi I. Perceived Neighborhood Social Cohesion and Preventive Healthcare Use. Am J Prev Med. 2017;53(2):e35–e40. doi: 10.1016/j.amepre.2017.01.007 28214249 PMC5522638

[29] Cárdenas D, Orazani N, Manueli F, Donaldson JL, Stevens M, Cruwys T, et al. Social cohesion predicts COVID-19 vaccination intentions and uptake. Social and Personality Psychology Compass. 2023;17(7):e12759.

[30] Qin C, Liu Q, Du M, Yan W, Tao L, Wang Y, et al. Neighborhood social cohesion is associated with the willingness toward the booster dose of COVID-19 vaccines among the Chinese older population. Human Vaccines & Immunotherapeutics. 2022;18(6). doi: 10.1080/21645515.2022.2140530 36375815 PMC9746548

[31] Eshel Y, Kimhi S, Marciano H, Adini B. Partial Social Integration as a Predictor of COVID-19 Vaccine Rejection and Distress Indicators. Front Public Health. 2022;10:900070. doi: 10.3389/fpubh.2022.900070 35958848 PMC9360764

[32] Kang YW, Ko YS, Kim YJ, Sung KM, Kim HJ, Choi HY, et al. Korea Community Health Survey Data Profiles. Osong Public Health Res Perspect. 2015;6(3):211–7. doi: 10.1016/j.phrp.2015.05.003 26430619 PMC4551141

[33] Kim J, Park MJ. Multilevel Effect of Neighborhood Social Cohesion and Characteristics on Suicidal Ideation Among Korean Older Adults. Community Mental Health Journal. 2021;57(3):522–8. doi: 10.1007/s10597-020-00678-5 32676878

[34] Geng H, Cao K, Zhang J, Wu K, Wang G, Liu C. Attitudes of COVID-19 vaccination among college students: A systematic review and meta-analysis of willingness, associated determinants, and reasons for hesitancy. Human Vaccines & Immunotherapeutics. 2022; 18(5):1–13. doi: 10.1080/21645515.2022.2054260 35438612 PMC9235888

[35] Bremer D, Lüdecke D, von dem Knesebeck O. Social Relationships, Age and the Use of Preventive Health Services: Findings from the German Ageing Survey. Int J Environ Res Public Health. 2019;16(21). doi: 10.3390/ijerph16214272 31689892 PMC6862648

[36] Lee HY, Jang SN, Lee S, Cho SI, Park EO. The relationship between social participation and self-rated health by sex and age: a cross-sectional survey. Int J Nurs Stud. 2008;45(7):1042–54. doi: 10.1016/j.ijnurstu.2007.05.007 17658532

[37] Park HK, Chun SY, Choi Y, Lee SY, Kim SJ, Park EC. Effects of social activity on health-related quality of life according to age and gender: an observational study. Health Qual Life Outcomes. 2015;13:140. doi: 10.1186/s12955-015-0331-4 26361977 PMC4566195

[38] Park NS, Lee BS, Chiriboga DA, Chung S. Loneliness as a mediator in the relationship between social engagement and depressive symptoms: Age differences among community-dwelling Korean adults. Health Soc Care Community. 2019;27(3):706–16. doi: 10.1111/hsc.12687 30485596

[39] Zhaoyang R, Sliwinski MJ, Martire LM, Smyth JM. Age differences in adults’ daily social interactions: An ecological momentary assessment study. Psychol Aging. 2018;33(4):607–18. doi: 10.1037/pag0000242 29708385 PMC6113687

[40] Ma M, Shi L, Liu M, Yang J, Xie W, Sun G. Comparison of COVID-19 vaccine policies and their effectiveness in Korea, Japan, and Singapore. International Journal for Equity in Health. 2023;22(1):224. doi: 10.1186/s12939-023-02034-x 37864164 PMC10588018

[41] Ministry of Health and Welfare. South Korea Announces the Roadmap for Gradual Return to Normal [cited 2021 Oct 27]. Available from: https://www.mohw.go.kr/board.es?mid=a20401000000&bid=0032.

[42] Korea Disease Control and Preventive Agency (KDCA). Status of COVID-19 Cases and Vaccinations in South Korea. [cited 2022 Oct 28]. Available from: https://www.kdca.go.kr/board/board.es?mid=a20501020000&bid=0015&list_no=720988&cg_code=C01&act=view&nPage=1&newsField=.

