# Correction: The potential role of perceived neighborhood social cohesion on COVID-19 vaccination uptake among individuals aged 50 and older: Results from the Korean Community Health Survey

**DOI:** 10.1371/journal.pone.0360162

**Published:** 2026-10-06

**Authors:** Younhee Kim, Min Kyung Lim

Tables 1, 2 and S1 Table were uploaded incorrectly. Please see the correct Tables 1, 2 and S1 Table here.

## Supporting information

S1 Table Crude odds ratios for COVID-19 vaccination from the univariate analysis. (DOCX)

**Table 1:** Baseline characteristics of the participants.

	Total	50–64 years old	65 years old or older
	n (weighted %) or mean ± SE	n (weighted %) or mean ± SE	n (weighted %) or mean ± SE
Total	135,352 (100.00%)	66,244 (48.94%)	69,108 (51.06%)
**Sex**			
Male	59,901 (44.26%)	30,342 (45.80%)	29,559 (42.77%)
Female	75,451 (55.74%)	35,902 (54.20%)	39,549 (57.23%)
Age (yrs.)	63.65±0.04	56.66±0.02	73.76±0.03
**Residence**			
Urban	64,526 (47.67%)	36,498 (55.10%)	28,028 (40.56%)
Rural	70,826 (52.33%)	29,746 (44.90%)	41,080 (59.44%)
**Education level**			
<High school	67,958 (50.21%)	17,008 (25.67%)	50,950 (73.73%)
≥High school	67,282 (49.71%)	49,172 (74.23%)	18,110 (26.21%)
**Income (monthly)**			
<3 Korean million won	78,651 (58.11%)	24,674 (37.25%)	53,977 (78.11%)
≥3 Korean million won	56,701 (41.89%)	41,570 (62.75%)	15,131 (21.89%)
Income (Korean million won)	371.95 ± 1.6	465.88 ± 2.18	236.26 ± 1.42
**Household composition**			
Living with family members	110,591 (81.71%)	58,196 (87.85%)	52,395 (75.82%)
Living alone	24,761 (18.29%)	8,048 (12.15%)	16,713 (24.18%)
**Smoking status**			
Non–smoker	117,433 (86.76%)	54,059 (81.61%)	63,374 (91.70%)
Smoker	17,917 (13.24%)	12,184 (18.39%)	5,733 (8.30%)
**Alcohol consumption (times/month)**			
<1	88,718 (65.55%)	35,377 (53.40%)	53,341 (77.18%)
≥ 1	46,623 (34.45%)	30,864 (46.59%)	15,759 (22.80%)
**Physical activities**			
No	112,095 (82.82%)	52,981 (79.98%)	59,114 (85.54%)
Yes	23,069 (17.04%)	13,196 (19.92%)	9,873 (14.29%)
**Experience of depression**			
No	126,007 (93.10%)	61,906 (93.45%)	64,101 (92.75%)
Yes	9,326 (6.89%)	4,336 (6.55%)	4,990 (7.22%)
**Self–reported health**			
Fair or poor	88,934 (65.71%)	38,509 (58.13%)	50,425 (72.97%)
Good	46,415 (34.29%)	27,734 (41.87%)	18,681 (27.03%)
**Trust between neighbors**			
Trust	101,189 (74.76%)	46,233 (69.79%)	54,956 (79.52%)
No trust	34,123 (25.21%)	19,980 (30.16%)	14,143 (20.47%)
**Mutual assistance among neighbors**			
Assistance	82,774 (61.15%)	34,782 (52.51%)	47,992 (69.44%)
No assistance	52,558 (38.83%)	31,448 (47.47%)	21,110 (30.55%)
**Satisfaction with healthcare service in community**			
Satisfaction	104,356 (77.10%)	49,861 (75.27%)	54,495 (78.85%)
No satisfaction	30,988 (22.89%)	16,377 (24.72%)	14,611 (21.14%)
**Concern about infection**			
High	88,245 (65.20%)	43,018 (64.94%)	45,227 (65.44%)
Moderate	27,079 (20.01%)	14,817 (22.37%)	12,262 (17.74%)
Low or no	20,016 (14.79%)	8,406 (12.69%)	11,610 (16.80%)
**Concern about social stigma**			
High	102,472 (75.71%)	49,419 (74.60%)	53,053 (76.77%)
Moderate	17,234 (12.73%)	9,096 (13.73%)	8,138 (11.78%)
Low or no	15,610 (11.53%)	7,717 (11.65%)	7,893 (11.42%)
**Concern about economic damage**			
High	106,241 (78.49%)	50,847 (76.76%)	55,394 (80.16%)
Moderate	14,921 (11.02%)	8,013 (12.10%)	6,908 (10.00%)
Low or no	14,178 (10.47%)	7,384 (11.15%)	6,794 (9.83%)

Values are presented as unweighted numbers (weighted %)

**Table 2:** Vaccination status and the results of the complex sample multiple logistic regression analysis.

	50–64 years old		65 years old or older	
	Vaccination	aOR (95% CI) 1)	Vaccination	aOR (95% CI) 1)
	n (weighted %) or mean ± SE		n (weighted %) or mean (SE)	
Total	61,394 (92.81%)	–	65,798 (95.00%)	–
**Sex**				
Male	27,862 (92.24%)	1.000 (Reference)	28,154 (95.21%)	1.000 (Reference)
Female	33,532 (93.37%)	1.200 (1.095, 1.314) *	37,644 (94.82%)	1.038 (0.929, 1.160)
Age (yrs.)	56.76 ± 0.02	1.097 (1.085, 1.109) *	73.66 ± 0.04	0.965 (0.957, 0.973) *
**Residence**				
Urban area	33,993 (93.16%)	1.000 (Reference)	26,656 (95.03%)	1.000 (Reference)
Rural area	27,401 (91.44%)	1.191 (1.079, 1.313) *	39,142 (94.91%)	1.166 (1.039, 1.309) *
**Education level**				
<High school	15,811 (92.61%)	1.000 (Reference)	48,511 (94.93%)	1.000 (Reference)
≥High school	45,527 (92.86%)	1.166 (1.048, 1.297) *	17,240 (95.09%)	0.865 (0.764, 0.978) *
**Income (monthly)**				
<3 million won	22,358 (90.26%)	1.000 (Reference)	51,310 (94.75%)	1.000 (Reference)
≥3 million won	39,036 (93.87%)	1.652 (1.509, 1.809) *	14,488 (95.55%)	1.124 (0.985, 1.284)
**Household composition**				
Living with family members	54,180 (93.21%)	1.000 (Reference)	49,926 (95.07%)	1.000 (Reference)
Living alone	7,214 (89.27%)	0.800 (0.719, 0.891) *	15,872 (94.68%)	1.143 (1.006, 1.297) *
**Smoking status**				
Non–smoker	50,536 (93.56%)	1.000 (Reference)	60,421 (95.12%)	1.000 (Reference)
Smoker	10,858 (89.60%)	0.644 (0.580, 0.714) *	5,376 (93.69%)	0.594 (0.497, 0.710) *
**Alcohol consumption (times/month)**				
<1	32,572 (92.02%)	1.000 (Reference)	50,475 (94.31%)	1.000 (Reference)
≥ 1	28,819 (93.65%)	1.552 (1.424, 1.691) *	15,317 (97.09%)	1.837 (1.574, 2.144) *
**Physical activities**				
No	49,035 (92.66%)	1.000 (Reference)	56,116 (94.68%)	1.000 (Reference)
Yes	12,295 (93.50%)	1.122 (1.012, 1.243) *	9,571 (97.12%)	1.516 (1.258, 1.827) *
**Depressive expression**				
No	57,486 (93.03%)	1.000 (Reference)	61,187 (95.30%)	1.000 (Reference)
Yes	3,907 (89.85%)	0.748 (0.651, 0.860) *	4,595 (91.35%)	0.596 (0.515, 0.689) *
**Self–reported health**				
Fair or poor	35,430 (92.10%)	1.000 (Reference)	47,731 (94.32%)	1.000 (Reference)
Good	25,963 (93.75%)	1.202 (1.109, 1.303) *	18,065 (96.57%)	1.458 (1.291, 1.646) *
**Trust between neighbors**				
Trust	43,002 (93.18%)	1.017 (0.928, 1.115)	52,562 (95.57%)	1.200 (1.058, 1.362) *
No trust	18,365 (92.13%)	1.000 (Reference)	13,228 (93.48%)	1.000 (Reference)
**Mutual assistance among neighbors**				
Assistance	32,368 (93.17%)	1.045 (0.951, 1.147)	46,041 (96.07%)	1.491 (1.312, 1.695) *
No assistance	29,013 (92.60%)	1.000 (Reference)	19,751 (93.88%)	1.000 (Reference)
**Satisfaction with healthcare service in community**				
Satisfaction	46,343 (93.09%)	1.106 (1.004, 1.219) *	52,056 (95.30%)	1.306 (1.160, 1.471) *
No satisfaction	15,046 (91.65%)	1.000 (Reference)	13,740 (93.48%)	1.000 (Reference)
**Concern about infection**				
Concern	40,035 (93.20%)	1.046 (0.922, 1.186)	43,181 (95.14%)	0.893 (0.761, 1.049)
Moderate	13,677 (92.40%)	1.000 (0.871, 1.148)	11,741 (95.53%)	1.140 (0.938, 1.387)
No concern	7,679 (91.66%)	1.000 (Reference)	10,869 (93.81%)	1.000 (Reference)
**Concern about social stigma**				
Concern	46,123 (93.36%)	1.344 (1.186, 1.522) *	50,785 (95.49%)	1.598 (1.345, 1.900) *
Moderate	8,309 (92.04%)	1.156 (0.997, 1.340)	7,711 (94.71%)	1.277 (1.020, 1.599) *
No concern	6,951 (90.64%)	1.000 (Reference)	7,281 (92.40%)	1.000 (Reference)
**Concern about economic damage**				
Concern	47,217 (92.88%)	0.968 (0.851, 1.102)	52,955 (95.37%)	1.276 (1.079, 1.509) *
Moderate	7,400 (92.81%)	1.021 (0.866, 1.205)	6,528 (94.57%)	1.096 (0.878, 1.368)
No concern	6,777 (92.40%)	1.000 (Reference)	6,307 (92.91%)	1.000 (Reference)

Note: adjusted odds ratios (aORs) and 95% confidence intervals (CIs) were derived by complex sample multiple logistic regression analysis

* P.<0.05
