# Supplementary material for: The potential role of perceived neighborhood social cohesion on COVID-19 vaccination uptake among individuals aged 50 and older: Results from the Korean Community Health Survey
Source: PLoS One. 2024 Oct 22;19(10):e0312309. doi: 10.1371/journal.pone.0312309 (PMC11495590; doi:10.1371/journal.pone.0312309)
Supplement: S1 Fig — (PDF) [file pone.0312309.s001.pdf]

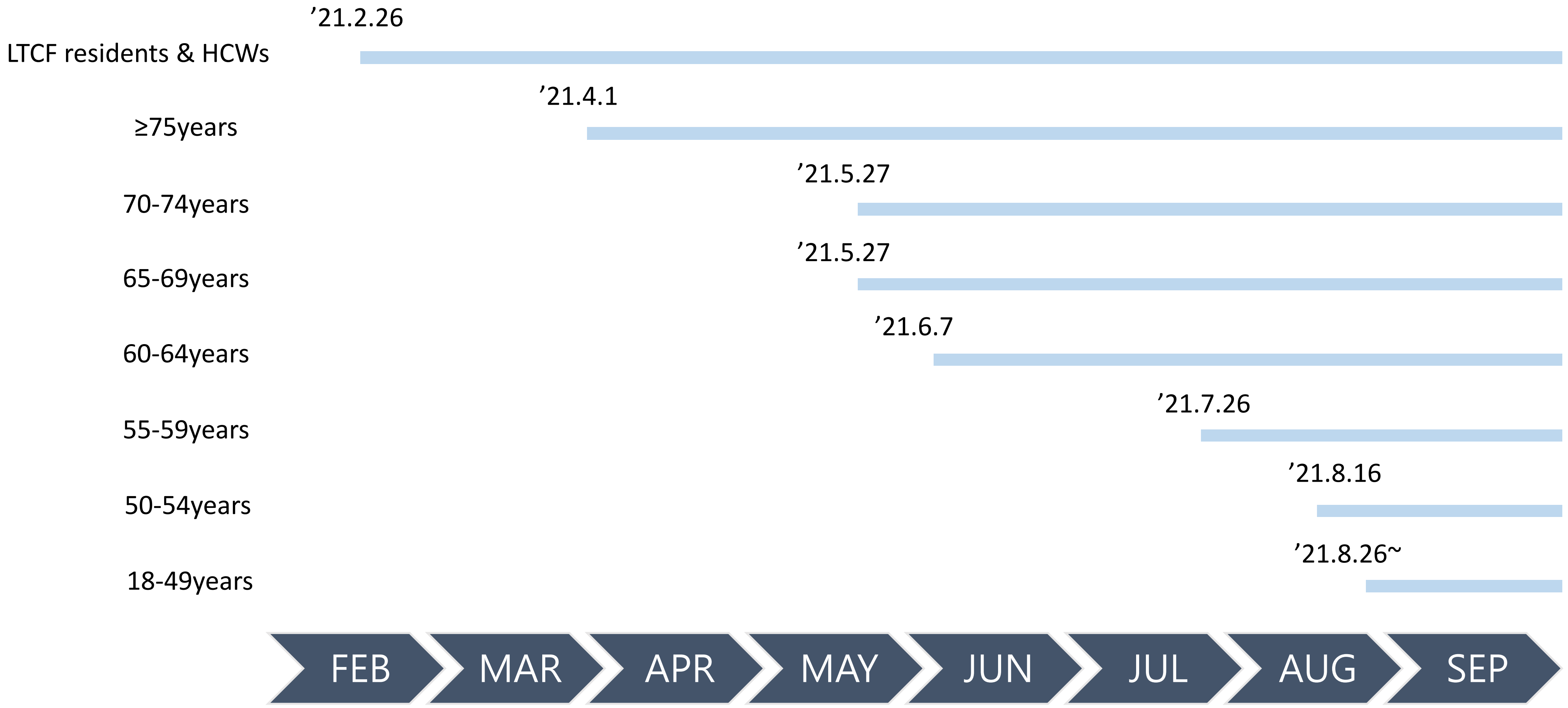

Supplementary Figure 1. Timeline of COVID-19 vaccination in South Korea

Note: LTCF = long-term care facilities, HCWs = healthcare workers.

Source: Korea Disease Control and Preventive Agency. Coronavirus Disease 19. Press release.
