# Supplementary material for: The potential role of perceived neighborhood social cohesion on COVID-19 vaccination uptake among individuals aged 50 and older: Results from the Korean Community Health Survey
Source: PLoS One. 2024 Oct 22;19(10):e0312309. doi: 10.1371/journal.pone.0312309 (PMC11495590; doi:10.1371/journal.pone.0312309)
Supplement: S1 Table — (DOCX) [file pone.0312309.s002.docx]

Supplementary Table 1. Crude odds ratios from the univariate analysis

|  | 50–64 years old | 65 years old or older |
| --- | --- | --- |
|  | cOR(95% CI) ^1)^ | cOR(95% CI) ^1)^ |
| Total | - | - |
| Sex |  |  |
| Male | 1.000 (Reference) | 1.000 (Reference) |
| Female | 1.185 (1.103, 1.273) ^*^ | 0.920 (0.841, 1.007) |
| Age (yrs.) | 1.076 (1.065, 1.086) ^*^ | 0.958 (0.950, 0.965) ^*^ |
| Residence |  |  |
| Urban area | 1.000 (Reference) | 1.000 (Reference) |
| Rural area | 1.278 (1.175, 1.390) ^*^ | 1.024 (0.932, 1.127) |
| Education level |  |  |
| <High school | 1.000 (Reference) | 1.000 (Reference) |
| ≥High school | 1.040 (0.947, 1.142) | 1.033 (0.928, 1.151) |
| Income (monthly) |  |  |
| <3 million won | 1.000 (Reference) | 1.000 (Reference) |
| ≥3 million won | 1.648 (1.522, 1.783) ^*^ | 1.189 (1.053, 1.342) ^*^ |
| Household composition |  |  |
| Living with family members | 1.000 (Reference) | 1.000 (Reference) |
| Living alone | 0.609 (0.551, 0.673) ^*^ | 0.925 (0.827, 1.034) |
| Smoking status |  |  |
| Non–smoker | 1.000 (Reference) | 1.000 (Reference) |
| Smoker | 0.761 (0.646, 0.896) ^*^ | 0.594 (0.545, 0.648) ^*^ |
| Alcohol consumption (times/month) |  |  |
| <1 | 1.000 (Reference) | 1.000 (Reference) |
| ≥ 1 | 1.281 (1.187, 1.383) ^*^ | 2.012 (1.736, 2.332) ^*^ |
| Physical activities |  |  |
| No | 1.000 (Reference) | 1.000 (Reference) |
| Yes | 1.141 (1.031, 1.262) ^*^ | 1.897 (1.578, 2.282) ^*^ |
| Depressive expression |  |  |
| No | 1.000 (Reference) | 1.000 (Reference) |
| Yes | 0.662 (0.579, 0.757) ^*^ | 0.521 (0.452, 0.600) ^*^ |
| Self–reported health |  |  |
| fair or poor | 1.000 (Reference) | 1.000 (Reference) |
| good | 1.291 (1.194, 1.397) ^*^ | 1.692 (1.503, 1.904) ^*^ |
| Trust between neighbors |  |  |
| Trust | 1.000 (Reference) | 1.000 (Reference) |
| No trust | 1.167 (1.075, 1.266) ^*^ | 1.506 (1.355, 1.673) ^*^ |
| Mutual assistance between neighbors |  |  |
| Assistance | 1.000 (Reference) | 1.000 (Reference) |
| No assistance | 1.089 (1.008, 1.177) ^*^ | 1.593 (1.450, 1.750) ^*^ |
| Satisfaction with healthcare service in community |  |  |
| Satisfaction | 1.000 (Reference) | 1.000 (Reference) |
| No satisfaction | 1.223 (1.117, 1.340) ^*^ | 1.412 (1.262, 1.581) ^*^ |
| Concern about infection |  |  |
| Concern | 1.245 (1.119, 1.385) ^*^ | 1.292 (1.145, 1.458) ^*^ |
| Moderate | 1.105 (0.974, 1.253) | 1.410 (1.204, 1.652) ^*^ |
| No concern | 1.000 (Reference) | 1.000 (Reference) |
| Concern about social stigma |  |  |
| Concern | 1.450 (1.304, 1.612) ^*^ | 1.739 (1.532, 1.973) ^*^ |
| Moderate | 1.192 (1.043, 1.361) ^*^ | 1.470 (1.237, 1.747) ^*^ |
| No concern | 1.000 (Reference) | 1.000 (Reference) |
| Concern about economic damage |  |  |
| Concern | 1.072 (0.955, 1.202) | 1.572 (1.374, 1.799) ^*^ |
| Moderate | 1.059 (0.906, 1.237) | 1.330 (1.103, 1.605) ^*^ |
| No concern | 1.000 (Reference) | 1.000 (Reference) |

Note: crude odds ratio (cORs) and 95% confidence intervals (CIs) were derived by complex sample simple logistic regression analysis

^*^ P<0.05
