# Supplementary material for: Correction: The potential role of perceived neighborhood social cohesion on COVID-19 vaccination uptake among individuals aged 50 and older: Results from the Korean Community Health Survey
Source: PLoS One. 2026 Oct 6;21(10):e0360162. doi: 10.1371/journal.pone.0360162 (PMC13641373; doi:10.1371/journal.pone.0360162)
Supplement: Supplementary file 1 [file pone.0360162.s001.docx]

**S1 Table. Crude odds ratios for COVID-19 vaccination from the univariate analysis.**

|  | **50–64 years old** | **65 years old or older** |
| --- | --- | --- |
|  | cOR (95% CI) 1) | cOR (95% CI) 1) |
| Total | - | - |
| **Sex** |  |  |
| Male | 1.000 (Reference) | 1.000 (Reference) |
| Female | 1.185 (1.103, 1.273) * | 0.920 (0.841, 1.007) |
| Age (yrs.) | 1.076 (1.065, 1.086) * | 0.958 (0.950, 0.965) * |
| **Residence** |  |  |
| Urban area | 1.000 (Reference) | 1.000 (Reference) |
| Rural area | 1.278 (1.175, 1.390) * | 1.024 (0.932, 1.127) |
| **Education level** |  |  |
| <High school | 1.000 (Reference) | 1.000 (Reference) |
| ≥High school | 1.040 (0.947, 1.142) | 1.033 (0.928, 1.151) |
| **Income (monthly)** |  |  |
| <3 million won | 1.000 (Reference) | 1.000 (Reference) |
| ≥3 million won | 1.648 (1.522, 1.783) * | 1.189 (1.053, 1.342) * |
| **Household composition** |  |  |
| Living with family members | 1.000 (Reference) | 1.000 (Reference) |
| Living alone | 0.609 (0.551, 0.673) * | 0.925 (0.827, 1.034) |
| **Smoking status** |  |  |
| Non–smoker | 1.000 (Reference) | 1.000 (Reference) |
| Smoker | 0.761 (0.646, 0.896) * | 0.594 (0.545, 0.648) * |
| **Alcohol consumption (times/month)** |  |  |
| <1 | 1.000 (Reference) | 1.000 (Reference) |
| ≥ 1 | 1.281 (1.187, 1.383) * | 2.012 (1.736, 2.332) * |
| **Physical activities** |  |  |
| No | 1.000 (Reference) | 1.000 (Reference) |
| Yes | 1.141 (1.031, 1.262) * | 1.897 (1.578, 2.282) * |
| **Depressive expression** |  |  |
| No | 1.000 (Reference) | 1.000 (Reference) |
| Yes | 0.662 (0.579, 0.757) * | 0.521 (0.452, 0.600) * |
| **Self–reported health** |  |  |
| fair or poor | 1.000 (Reference) | 1.000 (Reference) |
| good | 1.291 (1.194, 1.397) * | 1.692 (1.503, 1.904) * |
| **Trust between neighbors** |  |  |
| Trust | 1.167 (1.075, 1.266) * | 1.506 (1.355, 1.673) * |
| No trust | 1.000 (Reference) | 1.000 (Reference) |
| **Mutual assistance between neighbors** |  |  |
| Assistance | 1.089 (1.008, 1.177) * | 1.593 (1.450, 1.750) * |
| No assistance | 1.000 (Reference) | 1.000 (Reference) |
| **Satisfaction with healthcare service in community** |  |  |
| Satisfaction | 1.223 (1.117, 1.340) * | 1.412 (1.262, 1.581) * |
| No satisfaction | 1.000 (Reference) | 1.000 (Reference) |
| **Concern about infection** |  |  |
| Concern | 1.245 (1.119, 1.385) * | 1.292 (1.145, 1.458) * |
| Moderate | 1.105 (0.974, 1.253) | 1.410 (1.204, 1.652) * |
| No concern | 1.000 (Reference) | 1.000 (Reference) |
| **Concern about social stigma** |  |  |
| Concern | 1.450 (1.304, 1.612) * | 1.739 (1.532, 1.973) * |
| Moderate | 1.192 (1.043, 1.361) * | 1.470 (1.237, 1.747) * |
| No concern | 1.000 (Reference) | 1.000 (Reference) |
| **Concern about economic damage** |  |  |
| Concern | 1.072 (0.955, 1.202) | 1.572 (1.374, 1.799) * |
| Moderate | 1.059 (0.906, 1.237) | 1.330 (1.103, 1.605) * |
| No concern | 1.000 (Reference) | 1.000 (Reference) |

Note: crude odds ratios (cORs) and 95% confidence intervals (CIs) were derived by complex sample simple logistic regression analysis

* P<0.05
